# Internal femoral component rotation adversely influences load transfer in total knee arthroplasty: a cadaveric navigated study using the Verasense device

**DOI:** 10.1007/s00167-017-4640-5

**Published:** 2017-07-15

**Authors:** William A. Manning, Kanishka M. Ghosh, Alasdair Blain, Lee Longstaff, Steven P. Rushton, David J. Deehan

**Affiliations:** 1 Newcastle Surgical Training Centre Research Centre, Freeman Hospital, High Heaton, Newcastle upon Tyne, NE7 7DN UK; 2 School of Biology, University of Newcastle Upon Tyne, Newcastle upon Tyne, NE1 7RU UK; 3 Department of Orthopaedics, University Hospital of North Durham, Durham, DH1 5TW UK; 4 Institute of Cellular Medicine 4th Floor William Leech Building, Medical School, Newcastle upon Tyne, NE2 4HH UK

**Keywords:** Femoral rotation, Load, Flexion, Knee arthroplasty

## Abstract

**Purpose and hypothesis:**

Correct femoral component rotation at knee arthroplasty influences patellar tracking and may determine function at extremes of movement. Additionally, such malrotation may deleteriously influence flexion/extension gap geometry and soft tissue balancing kinematics. Little is known about the effect of subtle rotational change upon load transfer across the tibiofemoral articulation. Our null hypothesis was that femoral component rotation would not influence load across this joint in predictable manner.

**Methods:**

A cadaveric study was performed to examine load transfer using the orthosensor device, respecting laxity patterns in 6° of motion, to examine load across the medial and lateral compartments across a full arc of motion. Mixed-effect modelling allowed for quantification of the effect upon load with internal and external femoral component rotation in relation to a datum in a modern single-radius cruciate-retaining primary knee design.

**Results:**

No significant change in maximal laxity was found between different femoral rotational states. Internal rotation of the femoral component resulted in significant increase in medial compartment load transfer for knee flexion including and beyond 60°. External rotation of the femoral component within the limits studied did not influence tibiofemoral load transfer.

**Conclusions:**

Internal rotation of the femoral component will adversely influence medial compartment load transfer and could lead to premature polyethylene wear on the medial side.

## Introduction

Total knee arthroplasty (TKA) is designed to alleviate pain and restore function with optimal active range of movement. Contemporary primary knee replacement is met with 10-year survivorship in excess of 95%, up to 20% of the patient cohort remain less than entirely satisfied and early revision for loss of movement and pain is a significant clinical worry [1, 21, 35, 41]. In both registry and single-institution series, up to 20% of the patient group remain dissatisfied with the final outcome with greater than 50% of such cases related to poor movement or lack of stability and unexplained pain [4]. Modern designs of cruciate-retaining knee replacement systems aim to distribute load evenly across the articulating surfaces so as to both reduce polyethylene wear rates and ultimately the TKA revision burden [24]. The kinematic performance of the artificial knee is reliant upon exact placement with respect to the soft tissue envelope of the tibiofemoral and patellofemoral articulations [29]. The common denominator for both is the femoral component.

Femoral component placement may be performed using either a gap balancing or measured technique [26, 34]. No technique is consistently superior or reliable for femoral component placement [38]. Gap balancing is reliant upon correct tibial resection; otherwise, there is a compound error [14, 22]. Measured resection may introduce a malrotation due to difficulty identifying key anatomical landmarks [25, 43]. Femoral component malrotation will deleteriously influence the geometry of the flexion gap and patellar tracking [2, 17]. Load and constraint enjoy a complex and not always inverse relationship, and the flexion gap works with the soft tissue envelope and final laxity pattern to determine load across the flexed knee [8, 14]. Abnormal load may cause pain and stiffness. Our current standard biomechanical assessment is restricted to standing alignment views, and our knowledge of tibiofemoral load transfer in flexion is limited. A greater understanding of the load distribution across the tibiofemoral articulation under defined laxity conditions would allow for the study of the kinematic effect of component rotation and therefore subsequently predict the clinical performance of such a prosthetic joint [20].

In this study, we performed work to quantify the effect of femoral component rotation upon knee laxity and tibiofemoral contact force. Our primary (null) hypothesis was that femoral component rotation would neither influence load transfer across the tibiofemoral articulation nor maximal laxity for the knee arthroplasty construct at key points of knee flexion.

## Materials and methods

### Specimen demographics

**Fig. 1:**
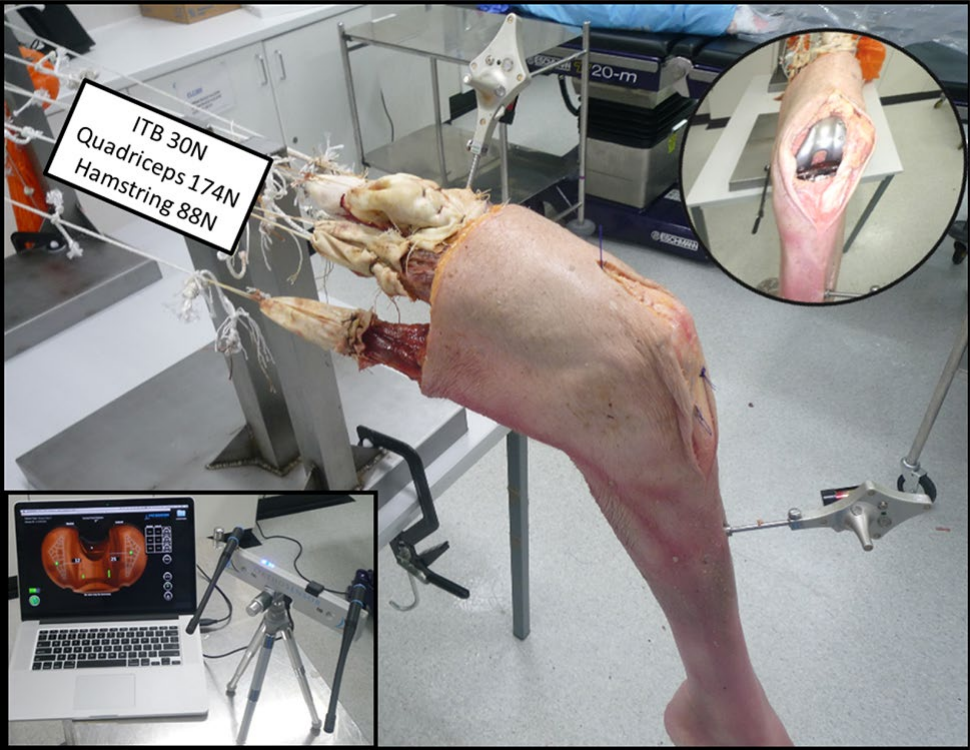
Experimental setup with custom jig and cadaveric limb loaded using a cable and pulley system to load in physiological directions. ITB 30 N, vastus lateralis 71 N rectus femoris and vastus intermedialis 61 N, vastus medialis 42 long and short head biceps femoris 44 N, semimembranous and semitendinosus 44 N. An arthrotomy has been performed (*inset* picture right) showing the Verasense tibial device is in situ with wireless hub connected to display unit (*inset* picture left). Navigation sensors on tibia and femur track motion and enabling laxity measurements

This work was performed under formal ethical approval and UK HTA licence within the surgical training facility xxxxxxxxxxx (Human Tissue Act 2004, section 16/2, licence number 12148). Whole lower limb cadaveric material without pre-existing radiographic evidence of malrotation/fracture/deformity or arthritic disease was used. Eight fresh frozen lower limbs disarticulated at the hip (three right, five left from Caucasian donors with median BMI 22, range 17–28, male/female ratio 7:1, and mean age 74, range 64–79 years) were prepared in a standardised manner as reported in previous published work [6, 9, 14]. Limbs were mounted onto a custom rig with the tibia hung vertically and secured using set screws to prevent specimen rotation. All muscle groups acting across the knee joint were loaded throughout the experimental procedure using previously validated methodology for the study of cadaveric knee kinetics. In particular, the iliotibial band (ITB), quadriceps muscle group, biceps tendon and medial hamstring apparatus were individually loaded in a physiological manner direction (Fig. 1). Navigation trackers (Stryker eNdtrac Knee Navigation System, Michigan USA) were fixed to the femur and tibia 25 cm from the joint line, respectively, and in such a manner as to avoid interfering with muscle pull [5]. Previous work had validated the use of eight limbs and confirmed that this provides sufficient power to identify significant differences using this technique with 95% confidence presuming 80% power [8, 22].

### Surgical procedure

**Fig. 2:**
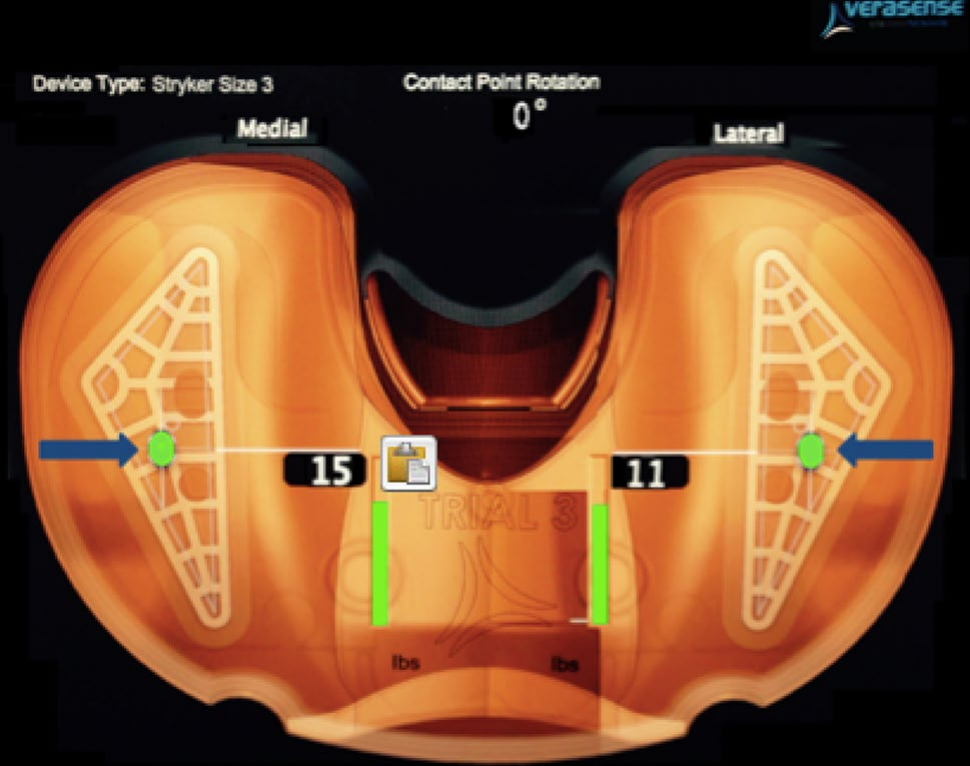
Verasense display as viewed by the surgeon during balancing. This image shows a rotationally matched tibiofemoral construct as well as a balance soft tissue envelope as defined by medial and lateral compartments contact forces the within 15lbs

**Fig. 3:**
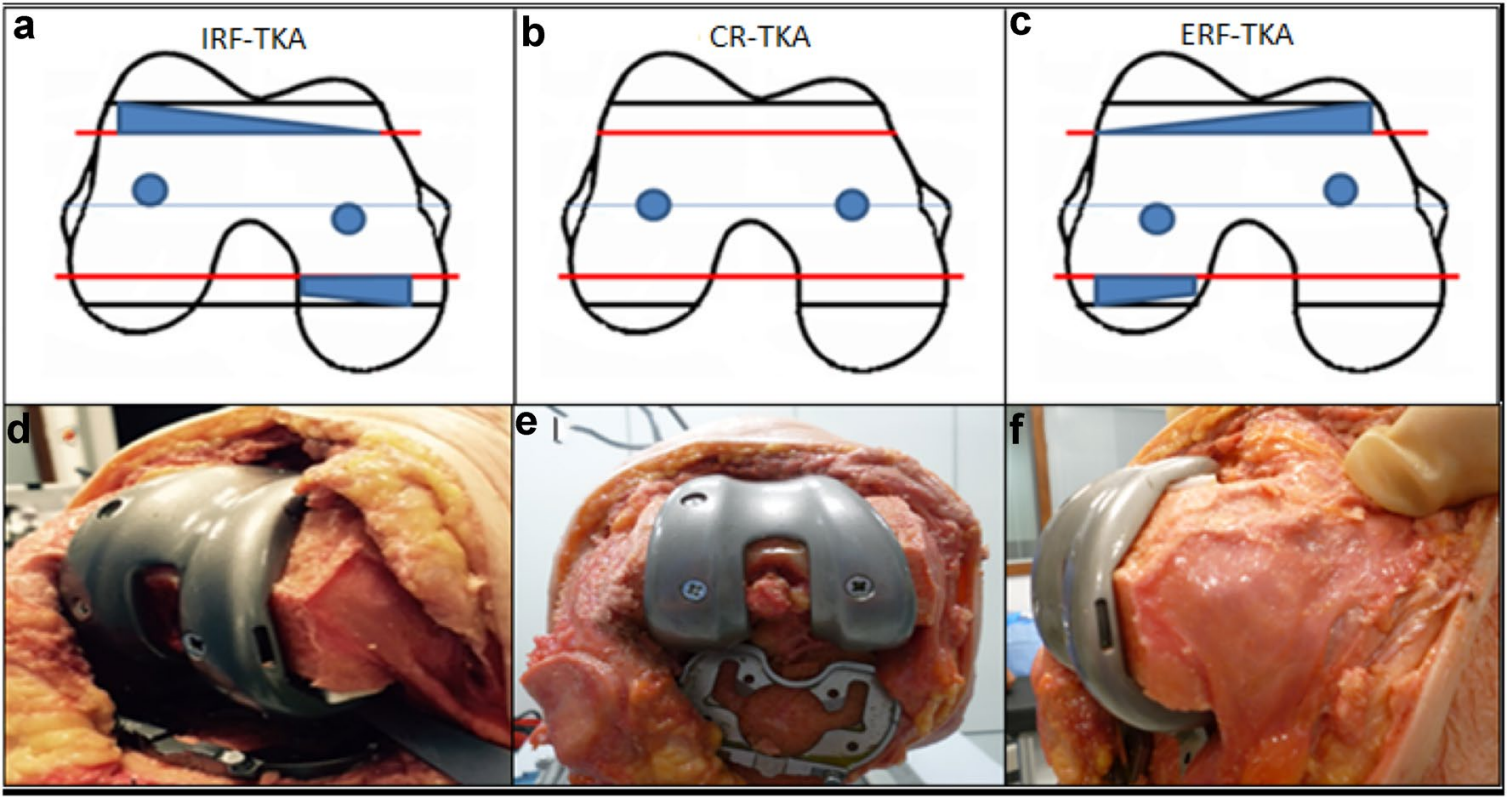
Pictorial representation of femoral component alignment, exaggerated for demonstration, showing the neutrally aligned CR-TKA (**b**), and the rotated femoral component to create the IRF-TKA (**a**) and ERF-TKA (**c**). *Black lines* demonstrating original femoral sizing and *red lines* demonstrating the downsized femoral cuts (2 mm) to accommodate for component rotation. Custom polyethylene *inserts* **d**, **f** provide reproducible rotation whilst maintaining correct femoral size and implant stability aided by lughole screws **e** for stress testing

Insertion of a Stryker Triathlon (Michigan USA) single-radius cruciate-retaining TKA (CR-TKA) via a medial parapatellar approach was undertaken using a measured resection technique [27, 34]. The femoral component rotation was determined using the transepicondylar axis, validated using the mid-trochlear axis. A balanced knee was confirmed as that where after minimal soft tissue release, there was passive full extension, full flexion with normal ‘no touch’ patellar tracking, less than grade 1 laxity for varus valgus stressing at 0°, 30°, 60° and 90° of flexion, firm endpoint to Lachman testing and no laxity for anteroposterior stressing at 90° of flexion. To achieve such, 8 mm of distal femoral resection was performed at 5° valgus intramedullary alignment. Femoral sizing was undertaken using anterior referencing. The mid-point of the medial epicondyle may be difficult to localise but to reduce any peroperative error both senior authors agreed the mid-point of such so as to define the epicondylar axis. The rotation of the final 4 in 1 femoral cutting block was further validated using the mid-sulcus or Whiteside line. The tibia was then prepared using a 9-mm resection from the superiormost tibial condyle in a plane perpendicular to the anatomical axis with an associated 3° posterior slope achieved via an extra-medullary jig. The tibial cutting block was centred at the mid-third of the tibial tubercle and formal resection performed. The trial tibial baseplate was centred without restraint on the cut surface. Final orientation of the tibial component was determined after cycling the knee >20 times. The final seated position which achieved greatest conformity was marked on the anterior tibial crest using diathermy. For each experiment and each position of femoral component rotation, the tibial component was allowed to find its most conforming position. A standard polyethylene insert was selected and inserted with trial components to ensure the flexion and extension gaps were stable for varus valgus and anteroposterior stressing at the defines points of flexion stated earlier. Full extension was confirmed. The flexion gap allowed for full flexion but was balanced such as to ensure no opening in deep flexion. So as to optimise femoral component stability on the distal femoral resection, lughole screws were inserted and captured the femoral component whilst being buried and not interfering in articulation with the poly-component. This ensured absence of component rotation or glide or translation at any point of the experimental stressing in 6° of motion during the flexion/extension arc of the experiment (Fig. 2). A Verasense (Orthosensor, Dania FL) of appropriate thickness then replaced the polyethylene insert. With the knee held in 10° of flexion, the tibial rotation was adjusted and secured when the Verasense system registered a parallel contact point rotation [12]. Medial and lateral tibiofemoral contact forces were recorded as the loaded knee was taken through a range of passive flexion without stressing. No procedure required more than a simple peri-articular medial or lateral capsular release to satisfy our criteria for balancing as defined by confirming the load across the medial and lateral compartments were within 15lbs (pounds-force) of each other, respectively, through a full arc of motion (Fig. 2) [12, 45]. This state defined the datum for femoral component rotation for each knee. To allow for ±3° of femoral rotation whilst maintaining the original component size, femoral cuts were downsized by 2 mm. The externally rotated (ERF-TKA) and internally rotated (IRF-TKA) states were achieved with the insertion of custom wedges (Fig. 3). The femoral component was fast secured following each rotation using custom cancellous thread lughole screws engaging the stronger subchondral bone. Care was taken to perform surgical closure of the arthrotomy via interrupted mattress sutures respecting the anatomy of the parapatellar tissues prior to any testing [31]. The senior authors (xxx/xx) performed all surgical procedures and stress testing. For all experiments, there were two senior surgeons, a surgical assistant, a manual operator of the orthosensor system, a senior scientist recording the output from the navigation system.

### Data capture and analysis

Data were captured, as per previous validated work, using a standard computerised navigation system with orthosensor provided range of compatible tibial trials [14, 37]. Knees were manually stressed to mimic intraoperative laxity assessment. A datum was taken from the knee in extension from which maximal displacements of the tibia in relation to the fixed femur were tracked via computer navigation (Stryker eNdtrac Knee Navigation System, Michigan, USA) to an accuracy of ±0.5 mm in 6° of freedom [5, 10, 28]. For each TKA condition, maximal displacements (anteroposterior, varus, valgus, internal and external rotation) were each recorded at five angles of flexion (0°, 30°, 60°, 90° and 110°). To reduce hysteresis, repeated flexion cycles were undertaken between measurements and ensured compartment forces remained constant during passive flexion. After each set of measurements, the output instrumentation was reset at zero. The Verasense device recorded tibiofemoral contact force (lbs/force) and contact points continuously during testing (millimetre accuracy ±2 mm—C. Anderson, OrthoSensor, personal communication 03.08.2015) with additional data capture under maximal stress at each of the five angles of flexion. Three knee conditions were defined with the internal and external rotatory states compared with the neutral or datum. These were the internally rotated femoral component (IRF-TKA) and the externally rotated femoral component (ERF-TKA) (Fig. 3).

### Statistical analysis

Mixed-effect modelling was used to quantify the effect of flexion angle, direction of movement and implantation of TKA upon laxity [30, 32]. Displacements were used as the response variable, with TKA and flexion as covariates. Student’s *t* test was used to compare differences in tibiofemoral force and contact point measurements. Significance was set at a level of *p* < 0.05.

## Results

### Native knee

**Fig. 4:**
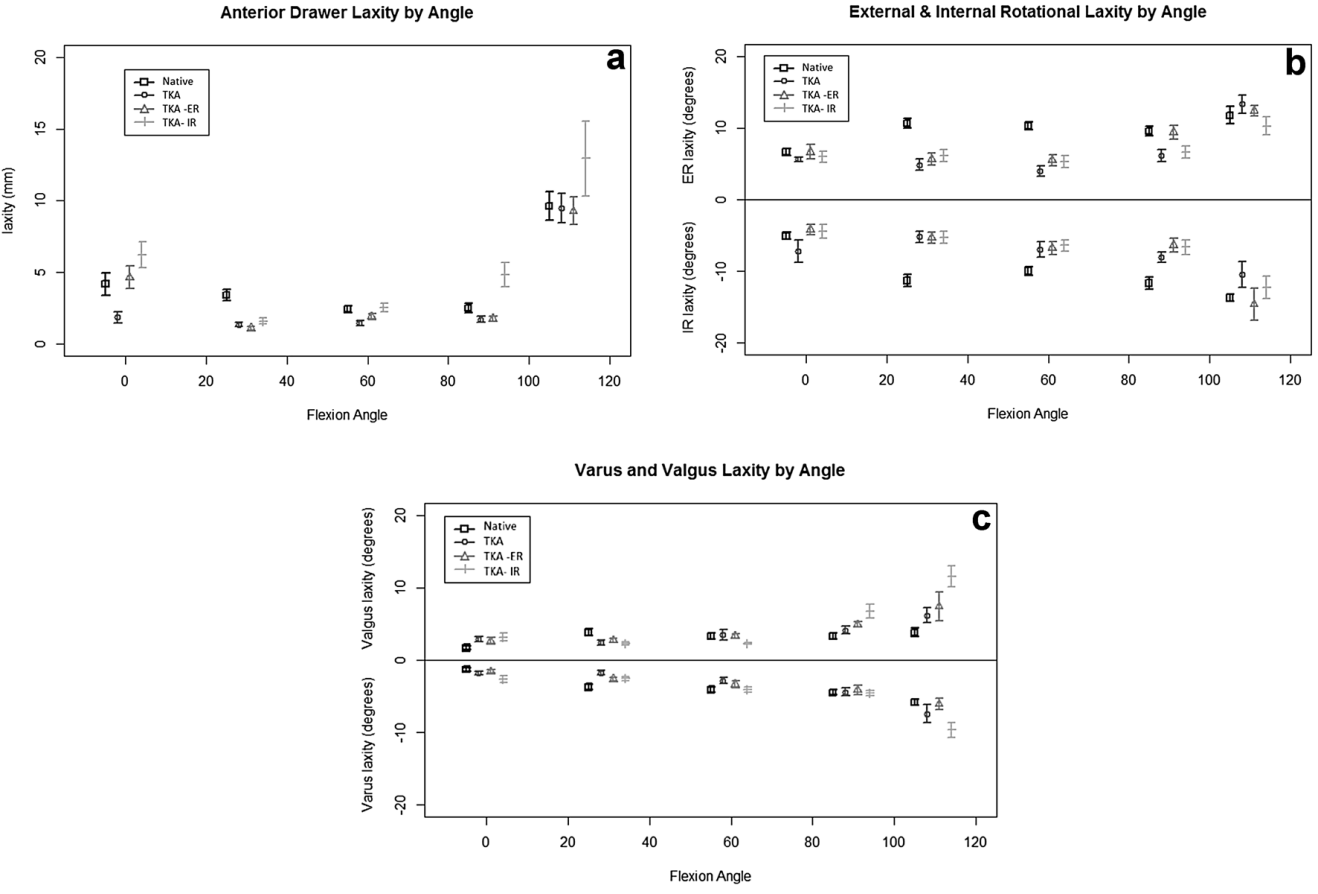
**a** Maximal anterior laxity (mm) under anterior draw stress testing with knee flexion (degrees). Native knee (*square*), CR-TKA (*circle*), ERF-TKA (*triangle*), IRF-TKA (*solid line*) *n* = 8. **b** Maximal rotational laxity (degrees) with knee flexion (degrees). Native knee (*square*), CR-TKA (*circle*), ERF-TKA (*triangle*), IRF-TKA (*solid line*) *n* = 8. **c** Maximal varus valgus laxity (degrees) with knee flexion (degrees). Native knee (*square*), CR-TKA (*circle*), ERF-TKA (*triangle*), IRF-TKA (*solid line*) *n* = 8

Total tibial rotation (combined internal and external rotation for each point of knee flexion of replaced state) and maximal varus/valgus laxity were found to increase consistently from full extension to deep flexion for all knee specimens. Rotational and coronal laxity did also increase with knee flexion within the native knee. This increase in laxity/rotation comparing the native versus the replaced state was significant for maximal anteroposterior movement (AD) when comparing 110°–30° (Fig. 4a).

### CR-TKA laxity pattern

A decrease in laxity was found for all knees after implantation of a single-radius (CR-TKA) cruciate-retaining knee design when compared to the native state. This reduction in maximal laxity only reached significance for rotatory laxity in higher levels of knee flexion beyond 90° (Fig. 4a–c).

### Load transfer

**Fig. 5:**
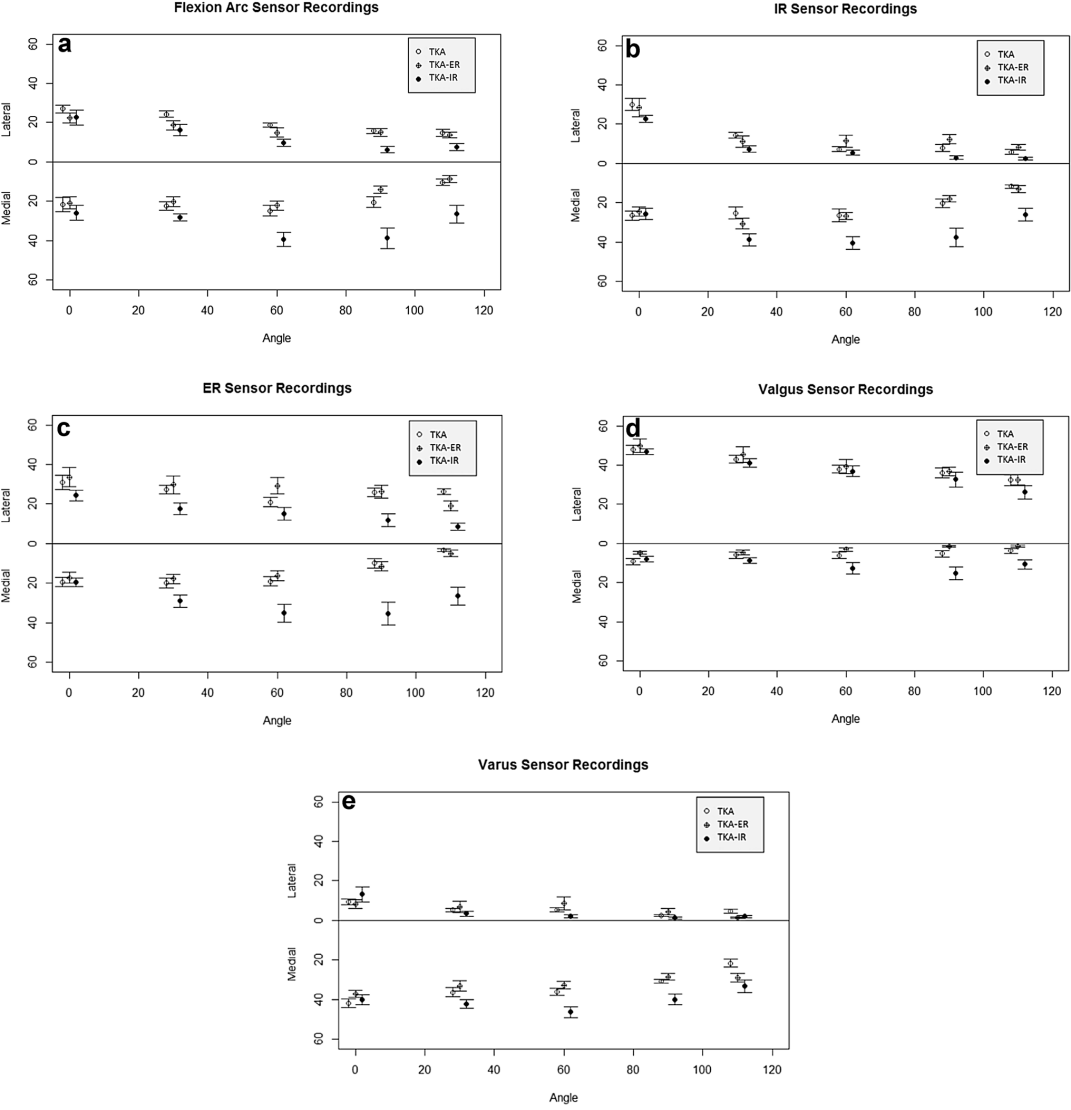
**a** Mean tibiofemoral contact force (lbs) during a normal flexion arc (degrees) with no external stress applied. CR-TKA (*clear circle left*), ERF-TKA (*crossed circle middle*), IRF-TKA (*solid circle right*) *n* = 8. **b** Mean tibiofemoral contact force (lbs) at maximal internal rotation stress with knee flexion (degrees). CR-TKA (*clear circle left*), ERF-TKA (*crossed circle middle*), IRF-TKA (*solid circle right*) *n* = 8. **c** Mean tibiofemoral contact force (lbs) at maximal external rotation stress with knee flexion (degrees). CR-TKA (*clear circle left*), ERF-TKA (*crossed circle middle*), IRF-TKA (*solid circle right*) *n* = 8. **d** Mean tibiofemoral contact force (lbs) at maximal valgus stress with knee flexion (degrees). CR-TKA (*clear circle left*), ERF-TKA (*crossed circle middle*), IRF-TKA (*solid circle right*) *n* = 8. **e** Mean tibiofemoral contact force (lbs) at maximal varus stress with knee flexion (degrees). CR-TKA (*clear circle left*), ERF-TKA (*crossed circle middle*), IRF-TKA (*solid circle right*) *n* = 8

Assessment of the unstressed flexion arc showed that contact forces across the medial and lateral compartments showed no statistical difference at each angle of flexion in the CR-TKA (Fig. 5a). Tibiofemoral contact force demonstrated equilibrium of load in the primary CR-TKA at each angle of flexion. The total force across the tibiofemoral joint was seen to decrease with flexion. An inverse relationship for total contact load versus maximal laxity was, as expected, found consistently for each knee.

### IRF-TKA laxity pattern

Maximal anteroposterior was significantly increased for the internally rotated femoral component state beyond 60° when compared to both the neutrally and externally rotated femoral component states. Varus valgus maximal movement and maximal rotatory motion assessment failed to identify any difference for the internally rotated component state when compared to neutral or external rotation (Fig. 4a–c).

### Load transfer

Load across the medial compartment was significantly increased at and beyond 60° of flexion with the internal femoral component state (Fig. 5b). This was most marked when stressing the knee with maximal internal rotation beyond 60° of flexion. At 90° flexion, a mean of 83% of the total contact load was borne by the medial compartment in the ERF-TKA compared to 47 and 43% in the CR-TKA and ERF-TKA, respectively (*p* < 0.05).

### ERF-TKA laxity pattern

The laxity pattern for the externally rotated femoral component knee arthroplasty (ERF-TKA) did not significantly alter when compared to the primary neutral position of the femoral component. Whilst the greatest difference between the ERF-TKA and CR-TKA was found at 110° with an increase in rotational laxity, this failed to reach statistical significance. No significant difference at any angle of flexion was found between the neutrally and externally rotated femoral component arthroplasty states for maximal anteroposterior movement or varus valgus stressing (Fig. 4a–c).

### Load transfer

Similar load was found across the medial and lateral femoral compartments between 0° and 30° of flexion when comparing the ERF-TKA versus CR-TKA states. Between 60° and 110° load increased across the lateral compartment under rotational stress for the ER-TKA when compared to the CR-TKA. This change did not, however, reach significance (Fig. 5c). It was representative of a proportional increase in lateral compartment loading in the ER-TKA state compared to the CR-TKA in mid to deep flexion. Overall, the ERF-TKA load pattern very closely resembled the pattern of contact force recorded for the neutrally aligned femoral component (CR-TKA) state.

### Load transfer with varus/valgus stress testing

There was consistency of load transfer across the medial and lateral compartments for both neutral datum femoral position and external rotation of the femoral component under both valgus (Fig. 5d) and varus maximal stress testing (Fig. 5e). Interesting, whilst this did not reach significance, there was a consistent increase in load transfer on the medial compartment in higher degrees of knee flexion for both varus and valgus load (Fig. 5d, e).

## Discussion

The principal findings of this work were the rejection of the null hypothesis that femoral component rotation would not influence load transfer across the tibiofemoral joint. Internal rotation of the femoral component led to increased load across the medial tibiofemoral compartment most markedly beyond mid-flexion. This redistribution of load was found even with a neutrally aligned knee replacement. It is possible that such increased load across the medial compartment could explain the pain reported by patients in clinical series with malrotated components [3, 17, 29]. The study failed to demonstrate a reciprocal increase in load on the lateral side with external rotation of the femoral component. Further, whilst the relationship between laxity patterns for total load across the tibiofemoral articulation mimicked those previous reported, the experiment failed to demonstrate a significant inverse relationship of such at the extremes of motion. Subtle internal malrotation of the femoral component resulted in a substantial increase in load transfer across the medial compartment. These differences were most evident for both varus and valgus stress testing in flexion. This dynamic biomechanical imbalance in flexion would not be detected in any standard long leg film view performed postoperatively and sheds light on the limitations of static views to determine biomechanical axes after surgery.

There are weaknesses inherent in the use of cadaveric material for such experimental work. None of the knees used in this work exhibited arthritic change or deformity. And whilst this does minimise the confounding effects of soft tissue contracture or bone loss, it may not entirely accurately replicate the operative state. However, the use of non-arthritic knees did allow for better accuracy when determining anatomical landmarks such as the epicondylar axis and ensured that baseline implantation was simple reliable and achieved quickly by the two senior operating surgeons. In contrast to previous work, all muscle groups acting across the knee joint were loaded as per previous methodology [22]. Particular attention was made to ensure free full tibiofemoral and patellar movement in the native loaded state and after primary implantation, cognisant of previous work, thereby confirming the importance of load across the joint to achievement of inherent stability [16, 22, 46]. The loads used in this study were subphysiological consistent with previous work so as to reduce risk of muscle tearing. Time zero assessment takes no account of the patient size, demographics or activity level. However, the use of cadaveric knees does allow for repeated-measures statistical analyses and reduces the confounding influence of pathological or functional differences between patients, thereby potentially enhancing the power of this work. Furthermore, prior to experimentation, all limbs had undergone radiographic assessment to exclude peri-articular deformity, degenerative change and confirmed neutral frontal plane alignment, which was confirmed as neutral for all limbs.

Excess internal femoral rotation in the absence of tibial malrotation is reported as an indication for revision arthroplasty [13, 23]. Several series confirm that revision for femoral component malrotation in isolation will achieve improved function with particular emphasis on functional measures which rely upon a stable and balanced mid-flexion tibiofemoral articulation [13, 23, 42]. Unlike the work of Thompson et al., we did allow for the tibia to sit on the tibial surface where there was maximal conformity, by cycling the knee after fixing the femoral component and allowing the tibia to sit accordingly, congruity was optimised from a load perspective [7, 33, 40]. Internal femoral component rotation with overstuffing of the medial compartment in flexion may stretch the MCL leading to pain, stiffness, failure to facilitate safe flexion and impaired varus/valgus laxity [11, 22, 33, 40]. Increased compartmental load on the medial side may lead to premature failure and suboptimal performance due to abnormal non-physiological load being shared across the articulation. Jeffcote et al. [15] correlated tibiofemoral forces with collateral ligament strain when increasing the flexion gap. Medial tibial pain has been reported in this patient cohort, and the finding of increased medial compartmental load with internal femoral component rotation could offer an explanation for such. We believe that this is the first work to identify abnormal preferential asymmetrical loading of the medial compartment without evidence of a reciprocal effect on laxity. Much previous work has argued that achievement of a quadrilateral flexion gap and the use of ligament tension devices may aid with load distribution but without determining load per se. However, this work does raise concerns about how reliable peroperative manual stress is at determining equivalent loading across the medial and lateral compartments [44]. Previous CT work has found rotational alignment errors in vivo to range from 13° of internal rotation to as much as 16° of external rotation have been documented [36]. Correct coronal plane alignment is associated with good clinical outcomes, but little is known of the correct mechanical axis in flexion [19]. Our work has focused on a much narrower range of rotation and despite such has still identified significant variation in load transfer. Greater errors in 3D placement can underscore the early clinical failures with patients reporting suboptimal functional performance from mediolateral pathological laxity and inevitable pain from loosening and abnormal loading of the subchondral bone [18].

Our work failed to find differences in load on the lateral side with external rotation of the femoral component. It is known that the posterolateral corner, additionally, is ineffective in flexion [39]. This apparent capacity to dissipate load in flexion could explain the minimal change in load on the lateral side with excess femoral external rotation. Recent work on the kinematics of femoral component rotation did examine the impact of femoral and tibial component rotation upon load in the surrounding muscle groups [40]. These workers found that femoral component rotation principally influenced load in the quadriceps apparatus and collateral ligaments, thereby determining varus valgus movement. However, no work was done to examine load across the joint. Our work has added to this knowledge by relating in isolation femoral component rotation, albeit with more subtle rotational margins [40].
